# A case of bilateral adrenal haemorrhage following traumatic brain injury

**DOI:** 10.1186/s40560-015-0073-8

**Published:** 2015-02-03

**Authors:** Mervyn Leong, Madhav Pendyala, Joga Chaganti, Suhel Al-Soufi

**Affiliations:** Intensive Care Unit, St Vincent’s Hospital, Sydney, Australia; Department of Radiology, St Vincent’s Hospital, Sydney, Australia

**Keywords:** Traumatic brain injury, Adrenal haemorrhage, Adrenal insufficiency

## Abstract

We report the case of a 57-year-old man who sustained an isolated severe traumatic brain injury (TBI). During his admission to the intensive care unit (ICU), he developed marked arterial hypotension of unclear cause. Eventually, the presence of renal angle tenderness on clinical examination and a low random-cortisol level lead to the suspicion of primary adrenal insufficiency. A computed tomography scan of his abdomen demonstrated new bilateral adrenal haemorrhages. This diagnosis is not unlikely to be missed, as symptoms and laboratory tests are often nonspecific.

## Background

Bilateral adrenal haemorrhages in the context of traumatic brain injury (TBI) are difficult to diagnose clinically despite appropriate symptoms and laboratory parameters as these are often nonspecific. Serum cortisol levels or the short synacthen test may not be specific for the diagnosis of primary adrenal insufficiency in critical ill patients, and there is a lack of data regarding normal cortisol levels in critical illness [1-4] Diagnosis can be made with computed tomography scan of the abdomen. We report a case of bilateral adrenal haemorrhages in an isolated TBI.

## Case presentation

A 57-year-old man sustained an isolated severe traumatic brain injury as a result of being struck as a pedestrian by a cyclist.

On admission to the emergency department, he had a Glasgow Coma Scale of eight, equal pupil size and reaction to light. He was hypertensive with a systemic blood pressure of 250/110 mmHg and in a sinus rhythm with 80 beats per minute. The patient was intubated for airway protection as he was vomiting profusely.

A computed tomography scan of the brain showed an acute right frontal subdural hematoma resulting in 1.1 cm of midline shift. In addition, there were bilateral fronto-temporal haemorrhagic contusions, diffuse subarachnoid blood along with right-sided parafalcine and uncal herniation. Whole-body computed tomography did not show any evidence of spinal, thoracic, abdominal or pelvic injury.

The patient was taken to theatres for right frontal decompressive craniectomy and evacuation of his subdural hematoma. An intra-parenchymal Codman catheter was inserted to monitor intra-cranial pressure.

Following his admission to our intensive care unit, the patient developed intracranial hypertension, which was controlled with deep sedation, continuous paralysis, targeted temperature management and infusion of hypertonic saline. He required in the first 4 days of his intensive care unit (ICU) admission infusion of noradrenaline in a dose of up to 0.25 mcg/kg/min and concomitant infusion of vasopressin at a dose of up to 0.01 U/min to maintain an adequate cerebral perfusion pressure. The random-cortisol level at this stage was judged to be adequate with 452 and 700 nmol/L at two subsequent days.

On day 6 of his ICU admission, following the cessation of vasopressors, he developed autonomic dysfunction with severe hypertension, which required temporary treatment with multiple enteral and intravenous anti-hypertensive medications.

In the second week of his admission to the ICU, the patient became again progressively hypotensive despite cessation of his anti-hypertensive medication. In the presence of low-grade fever, sepsis was suspected and antibiotics were empirically started. The patient had ongoing vasopressor requirements without response to intravascular volume replacement or a demonstrable cardiac cause. Cultures of sputum, blood and urine samples yielded no subsequent growth, and the white cell count remained normal.

**Figure 1:**
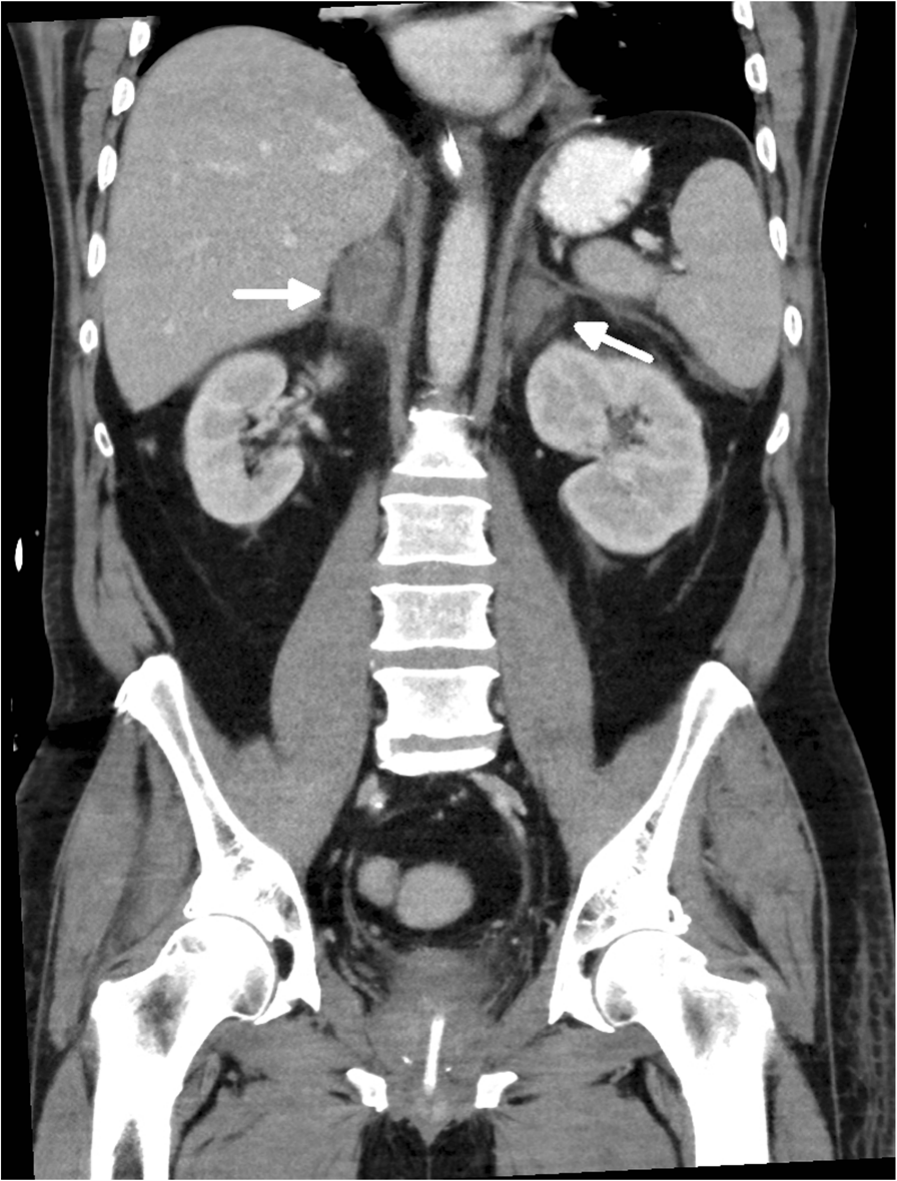
**Coronal contrast-enhanced scan.** Demonstrating enlarged bilateral adrenal glands of heterogeneous attenuation (average 40–50 U) (white arrow) with surrounding stranding.

**Figure 2:**
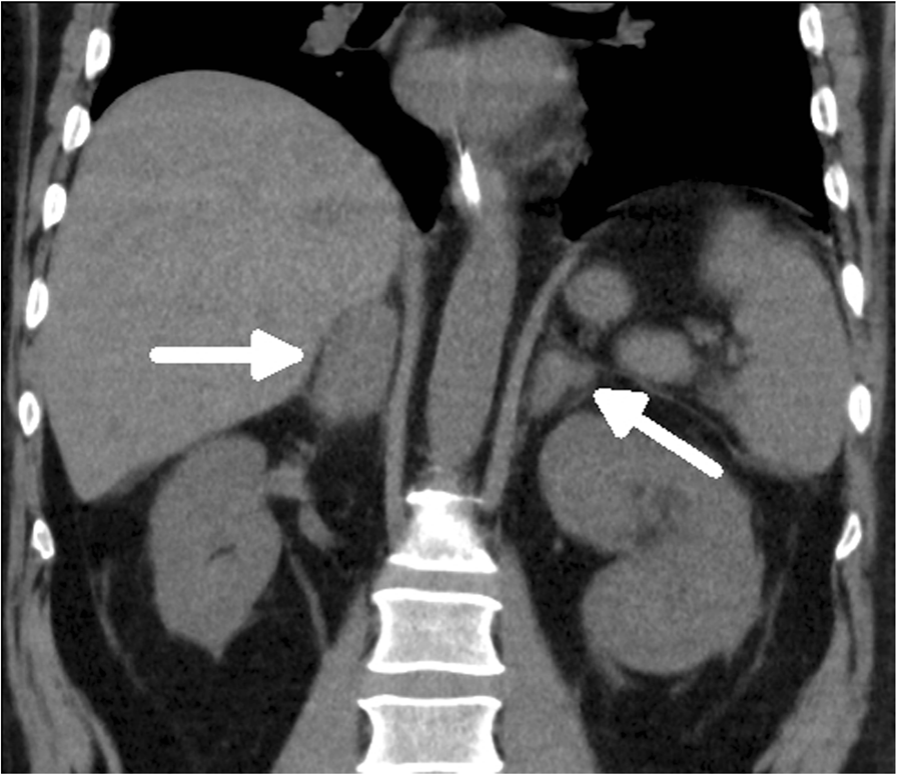
**Coronal noncontrast progress scan 4 days later.** Showing diffuse thickening of bilateral adrenal glands (white arrow) with ongoing stranding.

Eventually, primary adrenal insufficiency was suspected when renal angle tenderness was noted on clinical examination and a blood sample tested for random-cortisol showed a low level (116 nmol/L). A computed tomography scan of his abdomen demonstrated newly enlarged adrenal glands with heterogeneous attenuation and surrounding stranding which was highly suggestive of bilateral adrenal haemorrhages (Figure 1). The remainder of abdominal viscera appeared normal. The initial computed tomography scan was performed with contrast enhancement. However, a follow-up scan without contrast confirmed the diagnosis of bilateral adrenal haemorrhages (Figure 2).

Within 1 day of initiation of regular intravenous hydrocortisone 50 mg every 6 h, the hemodynamic status rapidly improved allowing the discontinuation of all vasopressors. Soon anti-hypertensive medication had to be recommenced.

Following discharge of this patient to the ward, hydrocortisone was reduced to oral 40 mg mane and 10 mg midi. Fludrocortisone 0.1 mg was added to the treatment to prevent episodes of orthostatic hypotension.

The patient was subsequently transferred to a rehabilitation centre where initially he had been progressing well with mobility and cognition. He represented eventually with postural hypotension, which lead to an increase of fludrocortisone to 0.15 mg.

## Discussion

Bilateral adrenal haemorrhages have been described in association with a host of clinical conditions such as septic shock (Waterhouse-Fredrickson syndrome) [5], disseminated intravascular coagulation, heparin-induced thrombocytopenia and thrombosis syndrome, antiphospholipid antibody syndrome, metastatic cancer, amyloidosis and granulomatous disorders. To our knowledge, there have been no reported cases of adrenal haemorrhage in the context of isolated severe traumatic brain injury.

We can only speculate on the nature of the association of traumatic brain injury and bilateral adrenal haemorrhage. After severe traumatic brain injuries, adrenal insufficiency can occur as a consequence of primary dysfunction of the hypothalamus or pituitary gland. However, the pathogenesis of adrenal haemorrhage is somewhat unclear. The anatomical blood supply of the adrenal glands is arranged in a way that the arterial blood supply is greater than the limited venous drainage capacity. Various factors can result in vascular engorgement and venous stasis within the adrenal glands. Stress-related ACTH secretion increases adrenal blood flow [6] while the catecholamine surge contributes to adrenal vein spasm. Platelet aggregation followed by venous thrombosis and disruption of the vascular endothelium further contribute to the development of intramedullary ischaemia and bleeding. We suspect that patients with severe traumatic brain injury who have high catecholamine levels and release tissue thromboplastin from damaged brain are at risk for bilateral adrenal haemorrhage to occur.

In the reported case, there was no evidence of any blunt trauma apart from isolated traumatic brain injury. The patient did not have a past medical history of malignancy or haematological disorder. Repeated coagulation profiles did not demonstrate any haematological abnormalities such as disseminated intravascular coagulation. The patient was not prescribed heparin or any other anticoagulant in the first weeks of his admission as this was considered to be contraindicated. We believe therefore that the bilateral adrenal haemorrhages were a consequence of the isolated traumatic brain injury.

Bilateral adrenal haemorrhages in the context of traumatic brain injury are difficult to recognise clinically, and the diagnosis might be missed despite dramatic symptoms. Abdominal or flank tenderness on examination of a patient with isolated severe TBI and shock should raise the suspicion of adrenal failure. Further suggestive though unspecific features are fever, anorexia, nausea and confusion, and in particular, hypotension or shock as are laboratory clues such as leukocytosis, hyperkalemia and hyponatremia in the presence of an unaccountable fall in haemoglobin [7].

Symptoms of adrenal insufficiency can be similar to that of the systemic inflammatory response syndrome and sepsis which are frequently seen in severe traumatic brain injury [8]. Adrenal insufficiency can be confused with other endocrine disorders related to TBI such as syndrome of inappropriate antidiuretic hormone secretion and cerebral salt wasting [2-4]. We believe that the lower than expected cortisol level in this patient is consistent with adrenal insufficiency caused by bilateral adrenal haemorrhages. However, serum cortisol levels or the short synacthen test may not be specific for the diagnosis of primary adrenal insufficiency in critical ill patients. Fluctuations in serum cortisol levels are frequent and not necessarily indicative of actual damage to the adrenal gland [1-4]. There is also a lack of consensus regarding normal cortisol levels in critical illness.

The diagnosis of adrenal haemorrhage can be made by a computed tomography scan of the abdomen demonstrating adrenal gland enlargement with high attenuation without contrast enhancement. Progress CT scans performed weeks after the acute phase may show progressive adrenal gland atrophy with variable degree of calcification. Ultrasonographic imaging of the abdomen may aid the diagnoses in the acute situation given that it is a bedside investigation that is easily accessible. Ultrasonographic examination usually reveals hyperechoic masses with central echogenic areas. Although magnetic resonance imaging may be helpful to exclude the presence of tumours and provide an estimate for the age of haemorrhage, there is very little evidence found in the literature.

In some cases, there may be spontaneous recovery even years after the original injury but patients with bilateral adrenal infarctions often require lifelong adrenal replacement therapy [9].

## Conclusions

This case demonstrates that bilateral adrenal haemorrhage can be a rare consequence of severe TBI. The diagnosis is difficult, as symptoms and laboratory test are often unspecific, but abdominal or flank tenderness on examination of a patient with isolated severe TBI and shock should raise the suspicion of adrenal failure and prompt appropriate imaging.

## Consent

Written and verbal consent was obtained from the patient and his next of kin for publication of this case report and use of accompanying images. A copy of the written consent is available for review by the Editor in Chief of this journal.
